# Multiparameter Neural Network Modeling of Facilitated Transport Mixed Matrix Membranes for Carbon Dioxide Removal

**DOI:** 10.3390/membranes12040421

**Published:** 2022-04-14

**Authors:** Rizwan Nasir, Humbul Suleman, Khuram Maqsood

**Affiliations:** 1 Department of Chemical Engineering, University of Jeddah, Asfan Road, Jeddah 23890, Saudi Arabia; kmaqsood@uj.edu.sa; 2 School of Computing, Engineering and Digital Technologies, Teesside University, Middlesbrough TS1 3BX, UK; h.suleman@tees.ac.uk

**Keywords:** neural network modeling, mixed matrix membranes, carbon dioxide removal, facilitated transport membranes

## Abstract

Membranes for carbon capture have improved significantly with various promoters such as amines and fillers that enhance their overall permeance and selectivity toward a certain particular gas. They require nominal energy input and can achieve bulk separations with lower capital investment. The results of an experiment-based membrane study can be suitably extended for techno-economic analysis and simulation studies, if its process parameters are interconnected to various membrane performance indicators such as permeance for different gases and their selectivity. The conventional modelling approaches for membranes cannot interconnect desired values into a single model. Therefore, such models can be suitably applicable to a particular parameter but would fail for another process parameter. With the help of artificial neural networks, the current study connects the concentrations of various membrane materials (polymer, amine, and filler) and the partial pressures of carbon dioxide and methane to simultaneously correlate three desired outputs in a single model: CO_2_ permeance, CH_4_ permeance, and CO_2_/CH_4_ selectivity. These parameters help predict membrane performance and guide secondary parameters such as membrane life, efficiency, and product purity. The model results agree with the experimental values for a selected membrane, with an average absolute relative error of 6.1%, 4.2%, and 3.2% for CO_2_ permeance, CH_4_ permeance, and CO_2_/CH_4_ selectivity, respectively. The results indicate that the model can predict values at other membrane development conditions.

## 1. Introduction

Carbon dioxide (CO_2_) is removed from natural gas streams to improve the calorific value of a product and to reduce corrosive damage to transmission pipelines. Conventionally, chemical absorption was used for CO_2_ removal, resulting in high capital and energy costs. New technologies, such as chemical looping [1], carbonate-based technology [2], membranes [3,4], cryogenics [5], adsorption [6], biology-based technology [7], and biobased ionic liquids [8], for carbon dioxide removal are continuously being developed and tested [3], with a goal to achieving better energy savings and waste minimization [9]. Membranes offer several advantages, e.g., low energy consumption, bulk separations, ease of operation, long service life, and minimal waste. The sieve-like structures in membranes separate different gases based on their kinetic diameters and, therefore, can reach theoretical purity nearing 100% [10,11]. However, in real-life applications, gases from a mixture may pass through a membrane depending upon the membrane’s permeability, selectivity towards each gas, and the gaseous mixture’s composition [11,12]. Hence, these parameters are significant during the membrane’s material selection and design.

Many membranes, such as anion-exchange membranes [13], hybrid membranes [14], polymeric membranes with nanoparticles [15], and graphene membranes [16], etc., have been developed to target gas separation. The “Robeson upper bound” evaluates the gas separation performance of membranes. Robeson’s upper bound relationship holds for various gas combinations, including CO_2_-CH_4_, O_2_-N_2_, H_2_-N_2_, H_2_-CH_4_, He-N_2_, He-H_2_, He-CO_2_, He-CH_4_, and H_2_-CO_2_ [17,18]. The separation of gases through membranes is based on the differences in gas diffusion rates. The diffusion rate depends on the differences in the gas molecules’ size, shape, and chemical nature. Typically, four types of membranes are used for gas separation: polymeric membranes, inorganic membranes [19], facilitated transport membranes (FTM) [20], and mixed matrix membranes (MMMs) [21]. Each of these membranes have different challenges in their development and use, and there are diverse perspectives on the development of various membrane materials [18]. Incorporating low molecular-weight additives (LMWAs) into the polymer matrix is favorable for membrane modification and improves its performance. Blending the polymer with LMWA is the most suitable approach (among others) for investigating the perm-selective and transport properties of membranes and the effect of LMWA type, nature, and concentration [22,23].

Researchers are developing various MMMs by incorporating third components: polymers and fillers. Miri et al. synthesized quaternary MMMs using Pebax@1657/glycerol/polyethylene glycol 200/iron-nickel oxide. They found that 3 wt.% of nanoparticle embedding was the optimum point that offered the best performance for CO_2_ permeability and CO_2_/CH_4_ and CO_2_/N_2_ selectivity [24]. Mohshim et al. investigated the impact of introducing ionic liquid into a flat sheet polyethersulfone-SAPO-34 mixed matrix membrane on CO_2_ separation performance. Optimal CO_2_/CH_4_ selectivity with the maximum ionic liquid concentration was found to be 62.6, compared to 20.7 for pure mixed matrix membranes. [25]. Shin et al. synthesized nanofiller-incorporated MMMs with high CO_2_ separation performances using CO_2_-philic polymers and 2D nanosheets. They used low molecular weight poly (ethylene glycol)–methyl ethyl acrylate as a free volume enhancer in the Pebax^®^ matrix and graphene oxide nanosheets as the nanofiller to improve both CO_2_ permeability and CO_2_/N_2_ selectivity. Long-term stability over 100 days was observed without a significant decline in separation performance [26].

The lab-scale analysis of the performance of MMMs could lead to the development of demonstrator/pilot units and to the later development of a commercial membrane system. For any such development, the lab-based research results must be correlated to the input (process) parameters, which can be reliably extended to industrial/end-user conditions without further experimentation [27]. Hence, correlative models are necessary, and many modeling approaches have been developed for MMMs. All models are based on ideal and non-ideal morphologies, as the separation performance is a strong function of the membrane morphology [28,29]. Numerous models based on their ideal and non-ideal morphology have been created to estimate the performance of MMMs [30,31,32]. Although the modeling approaches show excellent correlation and accuracy, they cannot correlate the multiple parameters required for evaluating membrane performance. For example, all modeling approaches can only correlate permeance for a single gas at one constant pressure. On the contrary, for successful membrane system development, a detailed comparison of the membrane’s permeability for different gases at different pressures is required. This helps to optimize membrane performance, finding the best set of operating conditions, selecting the best ratio among membrane casting materials, and reducing capital investment. None of the available models can perform this task. For example, for a pure gas, the membrane’s separation performance will depend on three parameters: permeance for gas A, permeance for gas B, and selectivity for both gases. These factors are affected by the materials used in casting the membrane and the pressure of each gas. These parameters are beyond the computational power of the conventional modeling approaches developed for membrane systems.

In recent years, neural network modeling of membrane processes has received enormous research interest. Feedforward neural networks are well-known instances of black-box models capable of accurately predicting the behaviors of complex surfaces and hybrid materials in a variety of chemical engineering processes [33,34]. Some studies have suggested that statistical correlation techniques such as kernel vector methods [35], partial–least square methods [36,37], and artificial neural networks (ANN) [34] could work for the non-linear experimental data in membrane technology. One study conducted a fault diagnosis of a polymer electrolyte membrane fuel cell system by using kernel vector methods [38]. Another study used the partial–least square method for the separation and semi-empirical modeling of ethanol–water solutions by pervaporation using a PDMS membrane. Among these techniques, ANN can correlate non-linear multi-parameter experimental data points containing high complexity [39]. Artificial neural networks are currently used in a wide range of membrane separation processes, including reverse osmosis, nanofiltration [40], ultrafiltration, microfiltration [41], gas separation [42], membrane bioreactors [43], and fuel cells [34]. The technique is based on a black-box model, and it employs the fundamentals of human brain learning to train, validate, and test developments in an agnostic manner [44].

This study proposes an artificial neural network modeling approach for the multiparameter correlation of facilitated-transport mixed matrix membranes. The model can theoretically predict a membrane’s permeability for all gases using only the material composition of the membrane and the pressure of each gas. The experimental permeance and the selectivity of a facilitated transport mixed matrix membrane developed for carbon dioxide removal from natural gas are correlated. The model optimizes the correlation for the above three parameters simultaneously, seeking inputs from the concentrations of the materials used in membrane casting and the pressure of each gas. The modeled results show an excellent agreement with the experimental values. They indicate that the model can be confidently used for predicting membrane performance (permeance and selectivity) for other conditions and for CO_2_/CH_4_ gas mixtures not tested during experimentation.

## 2. Membrane Synthesis, Characterization, and Gas Performance

**Figure 1:**
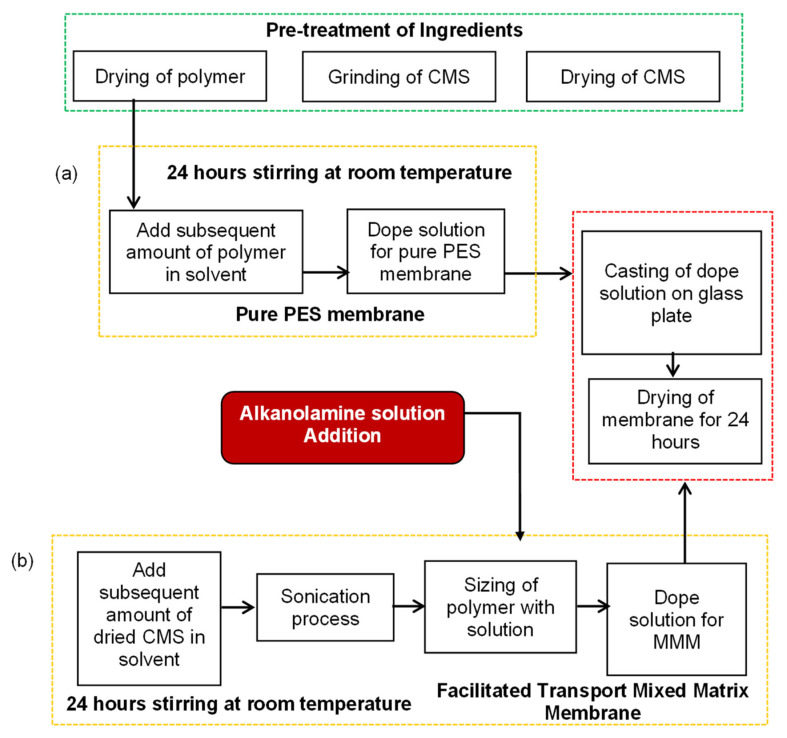
Synthesis flow diagram of (**a**) pure PES membrane, and (**b**) facilitated transport mixed matrix membranes.

The pure polymeric membranes and facilitated transport mixed matrix membranes were developed by using polyethersulfone (PES), a carbon molecular sieve (CMS) and diethanolamine (DEA). N-methyl-2-pyrrolidone (NMP) was used as a solvent. Membrane synthesis, characterization, and gas permeability were reported elsewhere [45,46,47]. The flowsheet of the membrane synthesis is shown in Figure 1. The components’ concentration and characterization techniques are provided in Supplementary Materials (Table S1 and Figure S1). The results of the gas performance analysis are also presented in the Supplementary Materials (Tables S2 and S3).

## 3. Methodology for ANN Development

**Figure 2:**
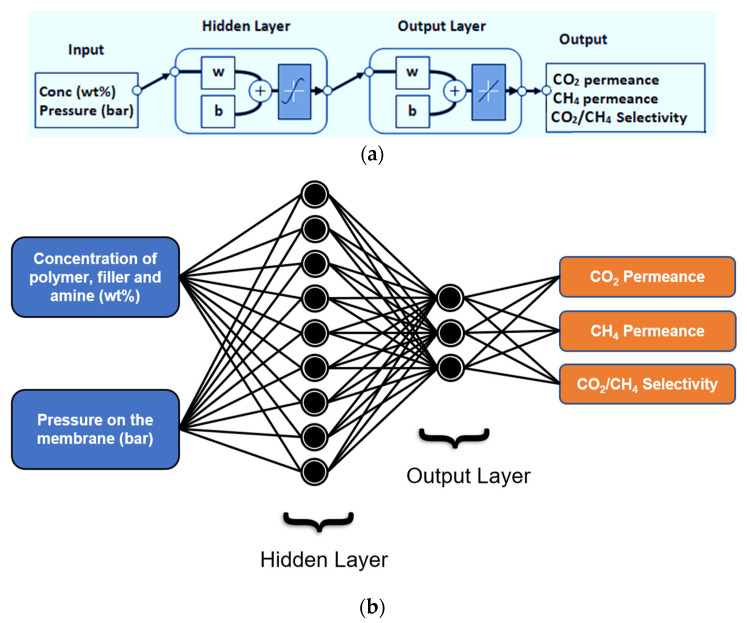
(**a**) Neural network typical design for proposed technique; (**b**) illustrative representation of neural network design.

The ANN for the predictive membrane model was developed in a MATLAB environment. The experimental data were collected from our previous work on single gas permeation through PES-CMS-DEA facilitated transport mixed matrix membranes, as discussed in Section 2 and Supplementary Materials. A typical two-layer feed-forward backpropagation type network was chosen due to its ease of design and distribution of experimental data in chemical streams [39,48], as shown in Figure 2. Different concentrations (wt.%) of the polymer (PES) (20 wt.%), 10–30 wt.% filler (CMS) (10–30 wt.%) and diethanolamine (DEA) (5–15 wt.%) and the pressure of the gas (2–10 bars) were inserted as data inputs to the neural network. Tangent sigmoid and linear transform functions were used for correlating parameters in the ANN’s hidden and output layers, respectively. Three target layers, i.e., CO_2_ permeance, CH_4_ permeance, and CO_2_/CH_4_ selectivity, were included in the developed ANN. Equation (1) was used to normalize all experimental data against the maximum value for each variable to minimize the effect of numerical values on the model and to avoid asymmetry in data correlation. The experimental data were distributed randomly in training (50%), validation (15%), and testing (35%) sets:

(1) $$Normalized\;\left(X\right)=\frac{X}{X_{max}}$$

where *X_max_* is the maximum value for variable *X*.

The number of neurons in the output layer was fixed at a value of 3 to correlate directly with the number of desired outputs, whereas the number of neurons in the ANN’s hidden layer was optimized [49]. The correlation is expected to improve with the increase in the number of neurons in the hidden layer, but the excessive number of neurons is also known to increase the complexity of the model and sometimes can reduce its accuracy [48]. In a typical optimization routine for the number of neurons, values from 1–30 were inserted in the ANN training mode [50] with an increment of 1 and were optimized using a minimum mean square error for all three sets (training, validation, and testing). Both Bayesian Coordinate and Levenberg–Marquardt algorithms were used. The latter was selected due to fewer iterations and better correlation (low mean square error (MSE) values). The following objective function was used to optimize the number of neurons in the hidden layer:

(2) $$MSE=argmin\frac{1}{n}\overset{n}{\underset{i=1}{\sum }}{\left(X_{o}-X_{T}\right)}^{2}$$

where *X_O_* and *X_T_* are the model’s output and the desired target values (experimental data) for each required output.

**Figure 3:**
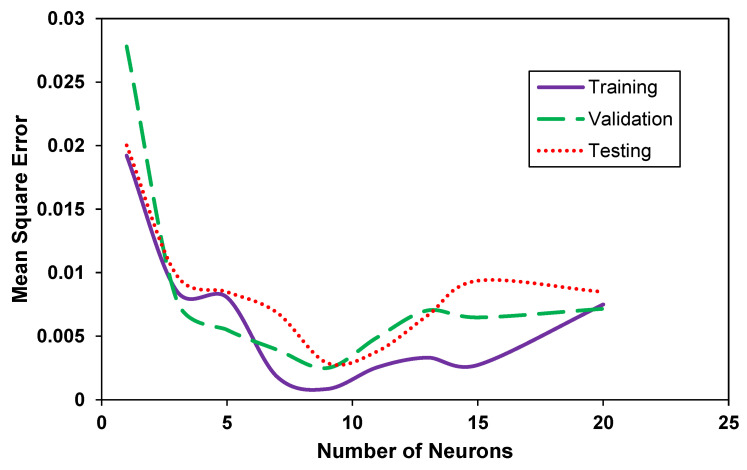
The change in the values of mean squared error against the number of neurons in the designed ANN.

Figure 3 shows the change in MSE values against the number of neurons in the ANN hidden layer. The MSE for all three distributed sets (training, validation, and testing) drops rapidly with an increase in neurons. Due to the highly non-linear nature of the data, a few peaks and troughs exist, showing poor correlation or overfitting of the data. For example, when the number of neurons is five, the training dataset shows a low correlation peak (with high MSE), while exhibiting a sudden trough at 15 (with low MSE). Correspondingly, the testing dataset (which is randomly distributed) shows a peak when the number of neurons is 15, reducing the overall MSE. Upon analyzing Figure 2, the lowest MSE for all three datasets is observed when the number of neurons is 9. Correlation models between gas pressure and material concentration for membrane development (and membrane permeance) were developed using this optimal value.

**Figure 4:**
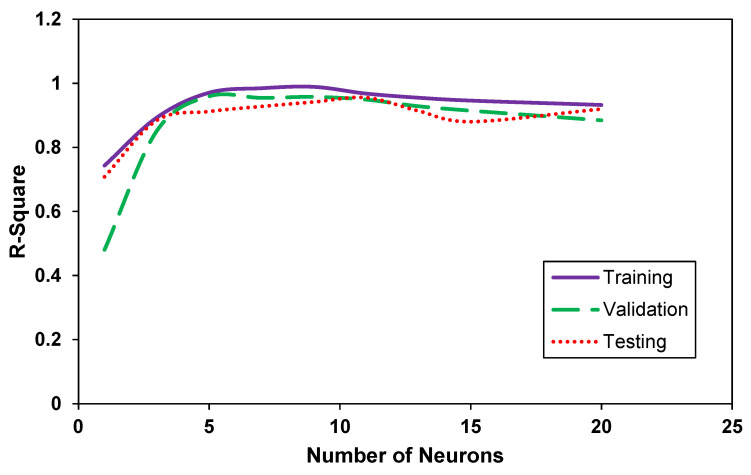
The change in the values of R-square against the number of neurons in the designed ANN.

Similarly, the R-square values of the model correlation were compared against the number of neurons. Figure 4 shows that the R-square value increases steadily from around 0.75 to 0.95 when the number of neurons is incrementally increased from 1 to 4. After this value, a plateau was observed, where the R-square values fluctuate minutely. Although the R-square value generally drops slightly as the number of neurons increases after a value of 10, this parameter cannot be used to pinpoint the optimal number of neurons but shows a general trend in terms of equal data distribution across a central plane. Hence, it serves as a guiding parameter for ascertaining the choice for the optimal number of neurons using MSE in Figure 3.

Once the number of neurons in the hidden layer was selected, the designed neural network was executed to determine its output values. The minimization of mean square error (MSE) in the experimental data pool was the objective function [51]. It is well known that neural networks are prone to over-fitting. This was avoided by checking the model’s correlation against linearity and controlled using the Mahalanobis distance [52,53] from the experimental values.

## 4. Results and Discussion

### 4.1. Effect of Pressure on the Permeance

**Figure 5:**
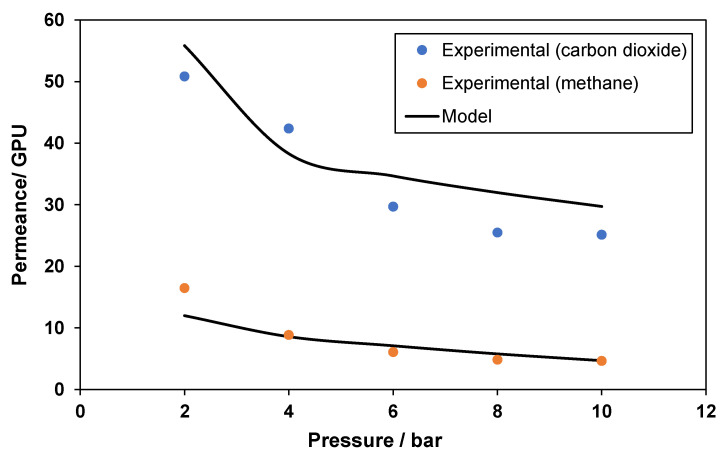
Model correlation for CO_2_ and CH_4_ permeances with respect to pressure for pure polymeric membrane.

The permeance of methane (CH_4_) and carbon dioxide (CO_2_), with respect to the pressure of each gas for a pure polymeric membrane, is shown in Figure 5. CH_4_ permeance is well correlated at all pressures except 2 bar. However, the modeled CO_2_ permeance does exhibit small deviations from all values. At a pressure of 2 bar, the trend is slightly over-predicted to counter the under-prediction of CH_4_ permeance, as both values are interlinked through the selectivity. The slight overprediction of the CO_2_ permeance value at a pressure of 4 bar can be attributed to experimental errors. The overall correlation for CO_2_ permeance is in line with the generic best fit observed for the rest of the experimental values for a pure polymeric membrane.

**Figure 6:**
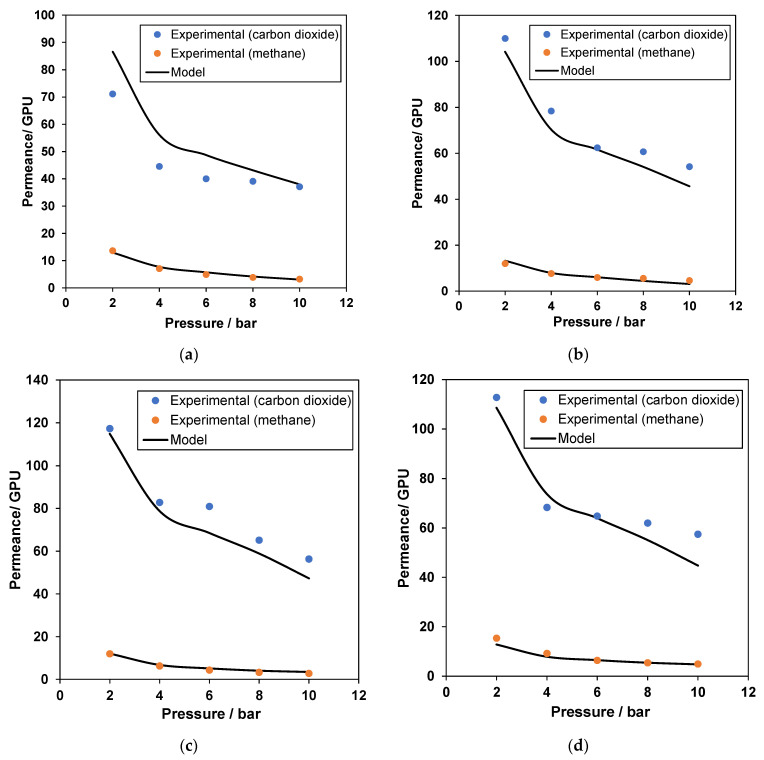

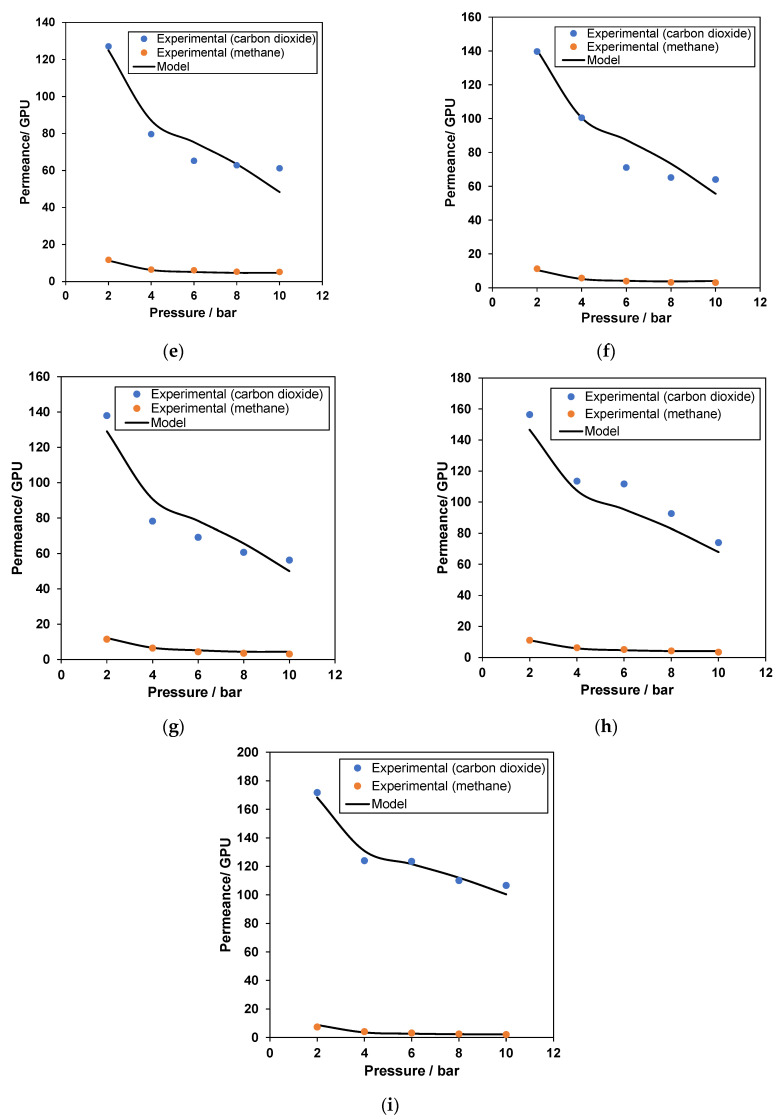
Model correlation for CO_2_ and CH4 permeances with respect to pressure for (**a**) 20% polymer, 10% filler, and 5% amine; (**b**) 20% polymer, 10% filler, and 10% amine; (**c**) 20% polymer, 10% filler, and 15% amine; (**d**) 20% polymer, 20% filler, and 5% amine; (**e**) 20% polymer, 20% filler, and 10% amine, (**f**) 20% polymer, 20% filler, and 15% amine; (**g**) 20% polymer, 30% filler, and 5% amine; (**h**) 20% polymer, 30% filler, and 10% amine; and (**i**) 20% polymer, 30% filler, and 15% amine (all percentages in weight percent). Experimental data from [45,46,47]. Detailed data are provided in the Supplementary Materials.

Figure 6a–i present the behavior of carbon dioxide and methane permeances with respect to changes in gas pressure for facilitated transport mixed matrix membranes that have polymer, filler, and amine. The overall correlation for CH_4_ permeance is in excellent correlation with the experimental values, whereas CO_2_ permeance shows significant variations. As the concentration of either the amine or the filler increases, the correlation for carbon dioxide improves considerably. The average absolute relative error for CO_2_ permeance is around ±8%, with maximum deviations up to +20%, mostly observed in Figure 6a. This can be attributed to the polymer’s glassy nature, which does not map equally with other components added to the membrane [54]. The decreasing trend of gas separation performance has been observed repeatedly elsewhere and is mostly due to the lower gas solubility coefficient at high pressures [55]. As seen in Figure 6b–i, the CO_2_ permeance values are slightly under-predicted at low pressure (2 bars), with nominal under-predictions at high-pressure conditions (10 bars). Nevertheless, the deviations seen in the model correlations are well distributed across the experimental values and are within the range of experimental error.

Interestingly, the model can chart the sudden drop in CO_2_ permeance at pressure values higher than 4 bars. This decrease in permeance at lower pressures stems from the gas’s decrease in solubility with increasing pressure. This follows the predicted behavior of the dual-mode sorption model, which is the combination of Henry’s law associated with the dense region, and Langmuir behavior, which is related to microvoids in the polymer [56]. Furthermore, when pressure increases, the contribution of the Langmuir area to overall permeability decreases, and gas permeability approaches a constant value associated with simple dissolution (Henry’s law) [57]. Moreover, the increases in CO_2_ permeance values with the addition of the amine and filler are well correlated by the model, with a decrease in AARE% values, which indicates the model’s capacity to predict CO_2_ and CH_4_ permeance at other conditions that are not tested experimentally.

### 4.2. ANN Predictability

**Figure 7:**
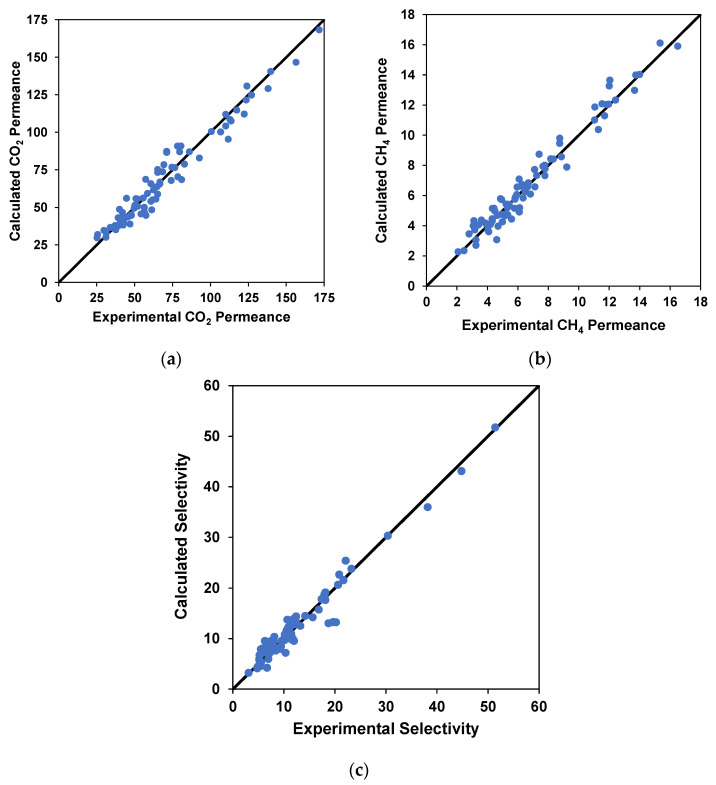
Parity plots for (**a**) calculated and experimental CO_2_ permeance, (**b**) calculated and experimental CH_4_ permeance, and (**c**) calculated and experimental selectivity for the experimental data.

The experimental results for CO_2_ and CH_4_ permeance and selectivity versus neural network predictions are plotted in Figure 7a–c. Most of the value points are satisfactorily close to the parity line, which presents a satisfactory fit of the modeled data to the real data. The coefficient of determination is more than 0.96 or greater for all cases, showing good agreement among the compared variables. This suggests that neural networks show high accuracy in the ANN model [58]. A similar agreement (R^2^ = 0.9997) between experimental and predicted values was found by Dashti et al., when they predicted the performance of nanocomposite membranes for H_2_ separation from CO_2_, C_3_H_8_, and CH_4_ by using artificial neural networks [59].

The model’s correlation can be further improved by adding another hidden layer that connects the first hidden layer to the output layer in the developed neural network model. However, such changes can impart a certain over-fitting to the results. Although over-fitted results are deemed good for experimental-based studies where the model’s results should show minimal errors, the results may not be viable enough to be extended beyond the range of regressed parameters. This study shows an artificial neural network-based approach for correlating CO_2_ and CH_4_ permeances and selectivity. Compared to the conventional models, this approach can crossmatch and optimize the results, while comparing three desired outputs for the best correlation. Although neural networks are not reproducible, we have tested the approach several times with a different range of parameters and normalized values and observed that the results are somewhat similar. We suggest that any future development in the field should accommodate linear variances and avoid over-fitting to the experimental values. The primary goal of a modeling approach is not to map the experimental values but to develop a good relationship that can predict values for non-tested conditions.

### 4.3. Relative Importance of Variables

Detecting effective variables on gas permeability is useful for designing, selecting, and managing gas separation membrane operations. To assess the strength of the link between output and input characteristics, the Cosine Amplitude Method (CAM) can be used. In this manner, all data pairings are stated in a general X-space [60]. The data pairs are applied to create a data array *X* as follows.

(3) $$X=\left\{X_{1},X_{2},X_{3},\ldots \ldots ,X_{m}\right\}$$

In the data array *X*, each of the elements (*X_i_*) is a vector of length of *m*.

(4) $$X_{i}=\left\{X_{i1},X_{i2},X_{i3},\ldots \ldots ,X_{im}\right\}$$

As a result, each data pair can be demonstrated by a specific location in m-dimensional space. Equation (4) provides the strength of the relationship between the data pairs *x_i_* and *x_j_*. The calculated *r_ij_* is a pairwise comparison of two X-space items [61].

(5) $$r_{ij}=\frac{\sum_{k=1}^{m}x_{ik}x_{jk}}{\sqrt{\sum_{k=1}^{m}x_{ik}^{2}\sum_{k=1}^{m}x_{jk}^{2}}}$$

**Figure 8:**
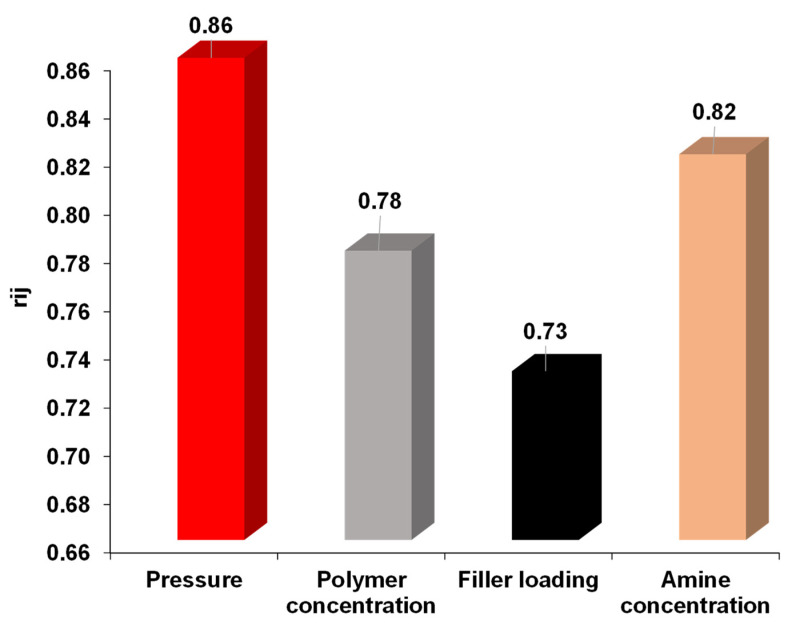
Sensitivity of process parameters for gas permeability through MMMs.

Figure 8 shows that of all the process input variables, pressure has the highest impact on the performance of developed membranes, followed by amine concentration, and then polymer concentration and filler loading. It was observed that gas permeance decreased with an increase in pressure, which is due to the polymer’s glassy nature. On the other hand, with the increase in amine concentration, CO_2_ permeance increases due to the affinity of CO_2_ with amine. Moreover, the sensitivity analysis results showed that pressure and amine concentrations are the most influential variables on gas permeability through facilitated transport mixed matrix membranes. These results are in agreement with the literature [60].

## 5. Conclusions

The current study presents a multiparameter modeling approach for simultaneously determining gas permeances and selectivity for a facilitated transport mixed matrix membrane. The model is flexible enough to accommodate any number of gaseous mixtures and their selectivity values. The inputs to the proposed model consist of the weight concentrations of the materials used for membrane casting and the gas pressure—giving the model an edge over classical modeling approaches that can only predict the permeance of a single gas at a single time—and correlated the values to it. Moreover, the model can be easily modified for different experimental data sets. The model results show an excellent correlation with the experimental values for a facilitated mixed matrix membrane designed for carbon capture. With an average absolute relative error of 6.1% for CO_2_ permeance, 4.2% for CH_4_ permeance, and 3.2% for CO_2_/CH_4_ selectivity, the model not only helps to correlate experimental data but is also capable of predicting intermittent values for membrane performance parameters for a CO_2_/CH_4_ feed mixture.

## Data Availability

Not applicable.

## Supplementary Materials

The following supporting information can be downloaded at: https://www.mdpi.com/article/10.3390/membranes12040421/s1, Figure S1: Systematic diagram of membrane characterization; Table S1: List of membranes synthesized in this study; Table S2: Experimental data; Table S3: Experimental data.
