# Supplementary material for: Multiparameter Neural Network Modeling of Facilitated Transport Mixed Matrix Membranes for Carbon Dioxide Removal
Source: Membranes (Basel). 2022 Apr 14;12(4):421. doi: 10.3390/membranes12040421 (PMC9028914; doi:10.3390/membranes12040421)
Supplement: Supplementary file 1 [file membranes-12-00421-s001.zip › membranes-1629843-supplementary.pdf]

Supplementary Information

Multiparameter neural network modelling of facilitated transport mixed matrix membranes for carbon dioxide removal

*Rizwan Nasir[1], Humbul Suleman[2], Khuram Maqsood[1],

[1]*Department of Chemical Engineering, University of Jeddah, Jeddah, Asfan Road, Saudi Arabia*

[2]*School of Computing, Engineering and Digital Technologies, Teesside University, Middlesbrough TS1 3BX, United Kingdom.*

*Corresponding author: E-mail: rnasir@uj.edu.sa

1 Membrane preparation schemes

Nine (09) different types of membranes were synthesized in this study. The list of membranes synthesized from this study is tabulated in Table S1.

Table S1: List of membranes synthesized in this study

Membranes	PES (wt. %)	CMS (wt. %)	DEA (wt. %)
Pure PES Membrane	20	-	-
Facilitated Transport Mixed Matrix Membrane	20	10	5
	20	10	10
	20	10	15
	20	20	5
	20	20	10
	20	20	15
	20	30	5
	20	30	10
	20	30	15

2 Characterization of Developed Membranes

Several characterization techniques were used to analyze the synthesized membranes. The characterization was carried out in four areas including surface and cross-sectional morphological, thermal, spectral and mechanical analyses were performed. Figure S1 shows the equipment used for the characterization.

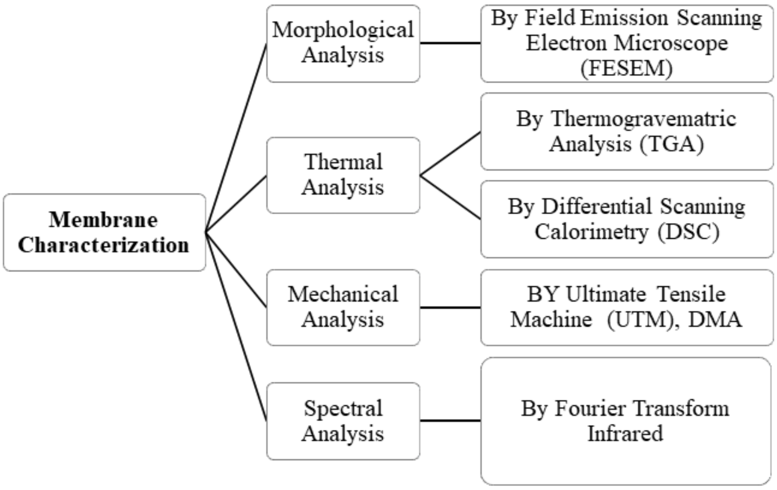

Figure S1: Systematic diagram of membrane characterization

Table S2: Experimental data

Pure Polymeric Membrane			
P (bar)	$(P/l)_{CO2}$ (GPU)	$(P/l)_{CH4}$ (GPU)	$\alpha_{CO2/CH4}$
2	50.87	16.49	3.08
4	42.41	8.86	4.79
6	29.71	6.08	4.89
8	25.51	4.86	5.25
10	25.14	4.65	5.40

GPU = Gas Permeation unit
P/l = Permeance
α = Selectivity

Table S3: Experimental data

Membranes	P (bar)	$(P/l)_{CO2}$ (GPU)	$(P/l)_{CH4}$ (GPU)	$\alpha_{CO2/CH4}$
MMM DEA 05% filler 10%	2	71.10	13.66	5.20
	4	44.55	7.09	6.28
	6	39.97	4.93	8.11
	8	39.11	3.85	10.15
	10	37.05	3.24	11.45

MMM DEA 10% filler 10%	2	109.98	11.99	9.17
	4	78.38	7.65	10.25
	6	62.40	5.91	10.55
	8	60.68	5.58	10.87
	10	54.15	4.60	11.76
MMM DEA 15% filler 10%	2	117.32	11.95	9.82
	4	82.81	6.26	13.22
	6	80.91	4.32	18.72
	8	65.18	3.32	19.62
	10	56.31	2.79	20.21
MMM DEA 05% filler 20%	2	112.80	15.34	7.36
	4	68.34	9.20	7.43
	6	64.82	6.39	10.14
	8	61.96	5.42	11.44
	10	57.46	4.92	11.67
MMM DEA 10% filler 20%	2	127.06	11.69	10.86
	4	79.63	6.47	12.30
	6	65.18	6.11	10.67
	8	62.85	5.33	11.79
	10	61.21	5.16	11.86
MMM DEA 15% filler 20%	2	139.66	11.28	12.38
	4	100.56	5.78	17.39
	6	71.10	3.92	18.14
	8	65.18	3.16	20.62
	10	63.99	3.07	20.84
MMM DEA 05% filler 30%	2	138.02	11.52	11.98
	4	78.21	6.49	12.06
	6	69.21	4.43	15.63
	8	60.68	3.61	16.83
	10	56.31	3.11	18.12
MMM DEA 10% filler 30%	2	156.42	11.03	14.18
	4	113.53	6.34	17.92
	6	111.73	5.16	21.65
	8	92.62	4.19	22.12
	10	74.09	3.41	21.71
MMM DEA 15% filler 30%	2	171.68	7.39	23.24
	4	123.92	4.08	30.36
	6	123.49	3.24	38.16
	8	109.98	2.46	44.79
	10	106.65	2.08	51.39

GPU = Gas Permeation unit
P/l = Permeance
α = Selectivity
